# Identification of cucurbitacins and assembly of a draft genome for *Aquilaria agallocha*

**DOI:** 10.1186/1471-2164-15-578

**Published:** 2014-07-09

**Authors:** Chuan-Hung Chen, Tony Chien-Yen Kuo, Meng-Han Yang, Ting-Ying Chien, Mei-Ju Chu, Li-Chun Huang, Chien-Yu Chen, Hsiao-Feng Lo, Shih-Tong Jeng, Long-Fang O Chen

**Affiliations:** Institute of Plant and Microbial Biology, Academia Sinica, 128 Sec. 2, Academia Rd, Nankang, Taipei, 11529 Taiwan; Department of Bio-industrial Mechatronics Engineering, National Taiwan University, Taipei, 106 Taiwan; Department of Horticulture and Landscape Architecture, National Taiwan University, Taipei, 106 Taiwan; Institute of Plant Biology, College of Life Science, National Taiwan University, Taipei, 106 Taiwan; Department of Computer Science and Information Engineering, National Kaohsiung University of Applied Sciences, Kaohsiung, 811 Taiwan; Department of Computer Science and Information Engineering, National Taiwan University, Kaohsiung, 811 Taiwan; Center of Systems Biology, National Taiwan University, Taipei, 106 Taiwan

**Keywords:** Agarwood, Cucurbitacin, Aquilaria, Genome

## Abstract

**Background:**

Agarwood is derived from Aquilaria trees, the trade of which has come under strict control with a listing in Appendix II of the Convention on International Trade in Endangered Species of Wild Fauna and Flora. Many secondary metabolites of agarwood are known to have medicinal value to humans, including compounds that have been shown to elicit sedative effects and exhibit anti-cancer properties. However, little is known about the genome, transcriptome, and the biosynthetic pathways responsible for producing such secondary metabolites in agarwood.

**Results:**

In this study, we present a draft genome and a putative pathway for cucurbitacin E and I, compounds with known medicinal value, from *in vitro Aquilaria agallocha* agarwood. DNA and RNA data are utilized to annotate many genes and protein functions in the draft genome. The expression changes for cucurbitacin E and I are shown to be consistent with known responses of *A. agallocha* to biotic stress and a set of homologous genes in *Arabidopsis thaliana* related to cucurbitacin bio-synthesis is presented and validated through qRT-PCR.

**Conclusions:**

This study is the first attempt to identify cucurbitacin E and I from *in vitro* agarwood and the first draft genome for any species of Aquilaria. The results of this study will aid in future investigations of secondary metabolite pathways in Aquilaria and other non-model medicinal plants.

**Electronic supplementary material:**

The online version of this article (doi:10.1186/1471-2164-15-578) contains supplementary material, which is available to authorized users.

## Background

*Aquilaria agallocha* is one of the largest producers of agarwood, a valuable product derived from Aquilaria and Gyrinops trees. Agarwood-producing tree species have become endangered due to the deforestation of tropical forests and the international trade of agarwood has come under strict control with a listing in Appendix II of the Convention on International Trade in Endangered Species of Wild Fauna and Flora. The use of agarwood is prevalent in many cultures, particularly in the Middle East and Asia where it has been used for over a thousand years. In particular, the use of agarwood is prevalent in religious ceremonies, herbal medicine, and as fragrances for perfumes and aromatherapy.

The main compounds related to the medicinal properties of agarwood are terpenes and phenylethyl chromone derivatives [1–3] which can be highly variable in content and composition among different agarwood-producing tree species. Previous studies have focused on sesquiterpenes, the most abundant terpenes compound in agarwood [4, 5]. However, little is known about triterpenoids in agarwood. Terpenoid content is induced under biotic stress as an immune response to resist various pathogens and its derivatives have been shown to exhibit anti-microorganism and anti-tumour functions [6, 7]. A specific compound of interest, cucurbitacin, is produced to combat fungal and bacterial pathogens [8]. Cucurbitacins have previously been isolated in Chinese medicinal herbs and have been shown to have pharmacological effects [6]. In particular, cucurbitacin I is known to repress cancer cell motility by perturbing actin dynamics and has also been known to exhibit cytotoxicity against MDA-MB-468 human breast cancer cells from animal models and indirectly interrupt actin dynamics [9]. The study of anti-tumour compounds and related pathways is thus an important field in agarwood research.

In this study, we identified cucurbitacin E and I from *in vitro A. agallocha* agarwood and present a draft genome for *A. agallocha. In vitro* materials were used to perform this study due to the lengthy growth period of resinous material in this species as well as to avoid contamination from microorganisms. Importantly, this process is applicable to plant factories for large-scale production in the future. The DNA and RNA sequence data were obtained using Illumina HiSeq 2000 sequencing technology, from which we performed *de novo* genome assembly and gene annotation. We inferred a putative pathway for cucurbitacin E and I from the genomic and transcriptomic data in order to better understand these important medicinal compounds in agarwood. The transcripts discovered to be related to the cucurbitacin pathway, were validated through qRT-PCR. To the best of our knowledge, this is the first draft genome for any species of *Aquilaria* as well as the first study to identify cucurbitacin E and I in agarwood from *in vitro* materials.

## Results and discussion

### Agarwood contains high cucurbitacin content

**Figure 1:**
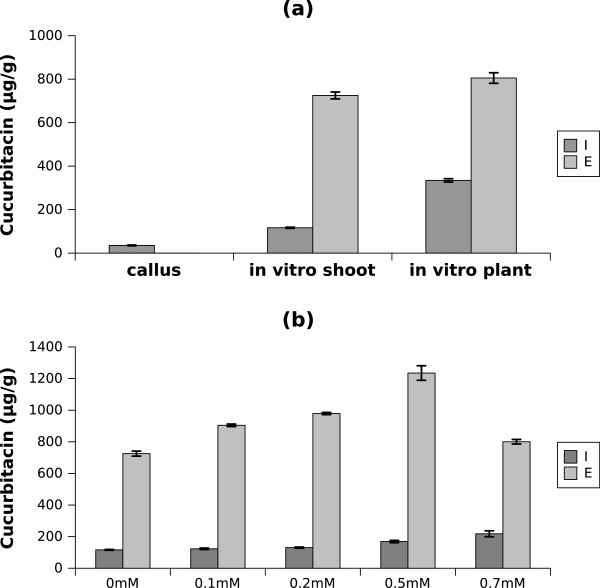
**Agarwood endogenous cucurbitacin E and I content. (a)** The endogenous cucurbitacin content was measured from different stages of agarwood plant. Data is represented as mean ± standard deviation (n = 5). **(b)** The endogenous cucurbitacin content of 2 month old agarwood *in vitro* shoot material was measured after treatment with various concentrations of methyl jasmonate (MJ). Data is represented as mean ± standard deviation (n = 5).

The strain of *A. agallocha* used in this study was originally derived from Myanmar and domesticated in Taiwan, after import. *In vitro* materials from this strain were analyzed using LC-ESI-MS where the presence of cucurbitacin I and E were detected (Additional file 1: Figures S1 and S2 respectively). After identification, *in vitro* callus, shoot and plant materials were analyzed for cucurbitacin E and I content (Figure 1a) where it was seen to be most abundant in *in vitro* plant. To the best of our knowledge, the cucurbitacin I content from *in vitro A. agallocha* produced agarwood is significantly higher than in any other Chinese medicinal herb studied previously, with an average concentration of 334.62 μg/g observed in this study as compared to previously reported concentrations of 0.55 μg/g and 25 μg/g from studies by Afifi et al. and Wu et al. respectively [10, 11].

As mentioned, agarwood produces terpenoids under biotic stress [8]. To investigate whether pathogens induce cucurbitacin content, the *in vitro* material was treated with methyl jasmonate (MJ), an important signal in pathogen related pathways. Various concentrations of MJ were applied to mimic different levels of biotic stress and the change in cucurbitacin content in response to different levels of biotic stress was measured. It was seen that cucurbitacin content increased as MJ concentration increased, up to 218 μg/g of cucurbitacin I at 0.7 mM MJ and 1,235 μg/g of cucurbitacin E at 0.5 mM MJ (Figure 1b). These results reveal that cucurbitacin pathway related genes were induced by MJ, indicating that agarwood may produce cucurbitacins and triterpenoids under biotic stress. With consideration for both cucurbitacin E and I peak values, the 0 mM and 0.5 mM MJ treatment samples from *in vitro* shoot were chosen for RNA-seq analysis.

### Assembly of a draft genome for *A. agallocha*

Although transcriptome assembly has become commonplace, the majority of transcripts obtained from transcriptome assembly are partial transcripts. Genome assembly may potentially allow for more complete transcript information to be obtained when compared to a *de novo* transcriptome procedure, thus easing primer design. As well, the sequence information of 5′ UTR regions is more easily obtainable from a genome assembly than from transcriptome studies. These factors allow for deeper studies of gene expression mechanisms.

The size of the haploid *A. agallocha* genome was estimated to be approximately 736 Mb by flow cytometry (Additional file 1: Figure S3). Five DNA libraries were constructed for a total of 144.3 Gb, which represents approximately 196X sequencing coverage (Table 1). The DNA libraries contained one pair-end library with a fragment length of 300 bp and four mate-pair libraries of various fragment lengths. For the *A. agallocha* transcriptome, two RNA libraries were constructed to represent MJ treatment conditions (0 mM and 0.5 mM MJ) where polyA + RNA from *in vitro* shoot was sequenced for a total of 12.5 Gb.

**Table 1:** **Sequencing read libraries for agarwood (** ***A. agallocha*** **)**

	Library	Type	Read length	No. read pairs
DNA	300 bp	paired-end	101 bp	384,629,812
	2 k bp	mate-pair	101 bp	55,940,209
	4 k bp	mate-pair	101 bp	47,800,413
	5 k bp	mate-pair	101 bp	109,363,782
	10 k bp	mate-pair	101 bp	116,549,154
RNA	0 mM MJ	paired-end	91 bp	40,919,476
	0.5 mM MJ	paired-end	91 bp	27,676,735

The DNA libraries were utilized in a *de novo* assembly procedure where the resulting draft genome (NCBI BioProject: PRJNA240626) contained 28,482 scaffolds with an N50 of 126.4 kb, a 1.3 Mb longest sequence, and a total size of 728.5 Mb, approximately 98% genome coverage (Table 2).

**Table 2:** **Summary of DNA pair-end libraries and** ***de novo*** **genome assembly**

Stage	N50 (kb)	Ave. (kb)	Total length (Mb)	Longest (kb)	No. sequences
Contigs	14.6	3.1	715.3	183.7	230,048
+2 k	43.8	7.7	716.1	517.2	92,539
+4 k	72.3	15.4	727.3	872.5	47,190
+5 k	94.0	20.2	727.5	995.7	36,097
+10 k	125.8	25.1	728.3	1289.8	29,057
Gapfilled	126.4	25.6	728.5	1291.6	28,482

### Gene annotation

The TIGR plant repeat database [12] and Repbase (2012/04/18) were combined with *ab initio* repeat prediction to perform repeat masking. This resulted in 59.18% of the draft genome classified as repeat sequences, with 27.57% classified as Long Tandem Repeat (LTR) elements. Transposable elements are generally non-coding DNA sequences that can change its location within a genome and can play an important role in development and evolution [13]. Thus, annotation of transposable elements was performed in this study using the TIGR plant repeat database (Table 3).

**Table 3:** **Repeated sequences annotation of transposable elements** ***via*** **TIGR database**

Class	No.	Size (bp)
Retrotransposon	747	142,807
Transposon	109	15,237
Miniature Inverted-repeat Transposable Elements (MITE)	1	62
Centromere-specific retrotransposon	1	57
Centromere satellite	10	778
Unclassified centromere sequence	8	1,770
Telomere sequence	11	2,215
Telomere associated	18	3,457
rDNA 45S	48	11,686
rDNA 5S	77	9,655
Unclassified (total)	361	38,797

**Figure 2:**
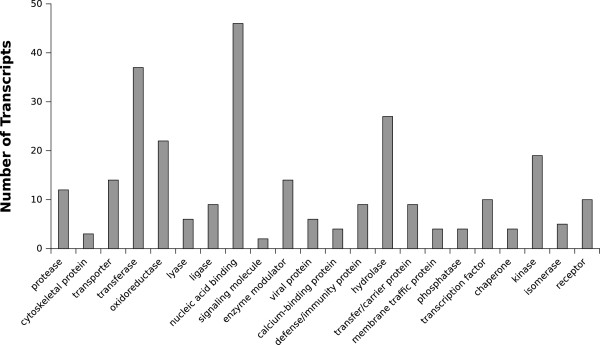
**Functional classification of annotated proteins.** The gene count for various protein classes as determined by Gene Ontology for the set of annotated proteins in *A. agallocha* agarwood.

RNA-seq data was aligned to the repeat-masked genome (achieving a mapping rate of 79.6%) to provide extrinsic support for gene prediction. *Ab initio* gene prediction combined with protein alignment resulted in annotations for 40,507 protein-coding genes, among which 3,257 genes encoded for multiple isoforms, representing 44,448 transcripts in total. On average, the predicted gene-models consisted of transcript lengths of 3,465.72 bp, coding lengths of 1,228.27 bp, and 5.48 exons per gene. A total of 66.7% of the predicted gene-models had matches in the NCBI non-redundant protein database, UniProt enzyme database, or matched a protein functional domain in Pfam. The transcriptome sequence data was able to be mapped to the draft genome at a mapping rate of 81.32%. As well, a total of 41.0% of gene-models were supported by 18,837 of 24,205 transcript sequences constructed from RNA-seq using the draft genome as a reference. A *de novo* assembled transcriptome was also aligned to the draft genome, where 33.0% of predicted exons were supported by 99,125 of 122,323 exons able to be mapped to the draft genome. Functional classification for the set of annotated transcripts was performed using Gene Ontology (GO) (Figure 2).

We compared our gene set to those from a recent study by Xu et al. [5], where a *de novo* transcriptome assembly for *Aquilaria sinensis* was performed. In their study, 38,159 transcripts were annotated with an average assembled sequence length of 678.65 bp as compared to our results of 44,448 annotated transcripts with an average length coding length of 1,228.27 bp. As well, 35,479 transcripts in our gene set contained both start and stop codons, which is another indicator for completeness. For gene discovery, assembling a draft genome clearly aids in the number of genes discovered as well as in the completeness of the assembled sequence.

### Differential expression

**Figure 3:**
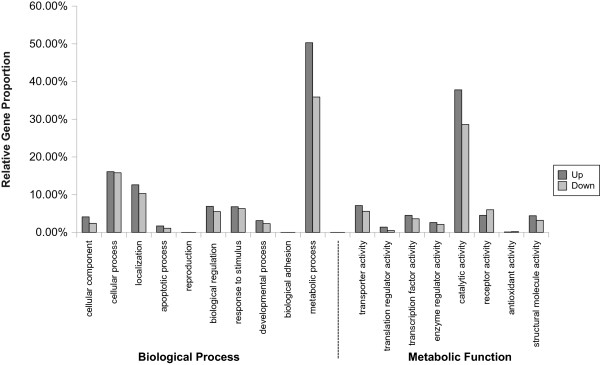
**Functional classification of differentially expressed transcripts.** Functional classifications of the set of differentially expressed transcripts through Gene Ontology, separated into up and down modes of regulation. It can be seen that metabolic processes and catalytic activity were enriched with up-regulated genes in the presence of biotic stress.

Under biotic stress and wounded conditions, MJ is an important signal in a plant’s defence system and will induce secondary metabolites. Thus, the set of annotated transcripts were analyzed for differential expression between the two treatment conditions, 0 mM and 0.5 mM MJ, in order to observe the effect of MJ on gene expression. The short reads from RNA-seq data were aligned to the set of annotated transcripts and the gene expression for each treatment condition was quantified, resulting in 4,827 differentially expressed genes (Additional file 2: Table S1) with at least a two-fold change in expression, of which 2,084 genes were up-regulated and 2,743 genes were down-regulated. Functional classification was performed for the set of differentially expressed genes, using GO (Figure 3). After treatment with 0.5 mM MJ, the activity of transcripts in the categories of metabolic processes and catalytic activity was observed to have increased, which is consistent with observations in previous studies [5].

### Putative pathway for cucurbitacin E and I

Cucurbitacins belong to the class of cytotoxic triterpenoids and are synthesized from MEP/DOXP and mevalonate pathways [8, 14]. Although the importance of medicinal compounds in *A. agallocha* agarwood is known, knowledge of its molecular mechanism is lacking and a transgenic line for this species is not able to be created. Thus, an assay of the cucurbitacin bio-synthetic pathway is currently not available.

**Figure 4:**
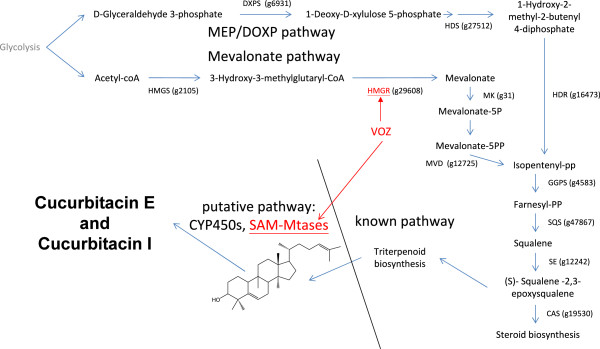
**Cucurbitacin E and I pathways in** ***A. agallocha.*** A schematic illustration showing cucurbitacin E and I bio-synthesis, including isoprenoid precursors via MEP and Mevalonate pathways. The *VOZ* transcription factor’s *cis*-element was observed to exist in *HMGR*.

To investigate the cucurbitacin pathway in *A. agallocha*, transcripts were annotated using *Arabidopsis thaliana* proteins as well as UniProt enzymes. The annotated transcripts were then used to infer a putative cucurbitacin pathway in *A. agallocha* by referring to the mevalonate pathway in *A. thaliana* from KEGG [15] as well as many differentially expressed cytochrome P450s (CYP450s) and S-adenosyl-L-methionine-dependent methyltransferases (SAM-Mtases) as putative downstream genes (Figure 4).

We identified genes which encode for enzymes in the cucurbitacin E and I pathway (Table 4), including three important gene categories: *HMGR* [16], *DXPS* [17], and *SQS* [17]; as well as genes which encode for synthases in cucurbitacin metabolism. These gene expression results are consistent with the knowledge that cucurbitacins are synthesized to resist pathogens [8]. We also investigated the vascular plant one-zinc-finger transcription factor (*VOZ*), described as follows.

**Table 4:** **Enzymes identified in the cucurbitacin E and I pathway and their change in gene expression**

Enzyme	Enzyme	0.5 mM/0 mM
Name	Commission	Ratio
1-deoxy-D-xylulose-5-phosphate synthase (DXPS)	2.2.1.7	7.1334
1-deoxy-D-xylulose-5-phosphate reductoisomerase (DXR)	1.1.1.267	1.2901
2-C-methyl-D-erythritol 4-phosphate cytidylyltransferase (MCT)	2.7.7.60	0.4569
4-diphosphocytidyl-2-C-methyl-D-erythritol kinase (CMK)	2.7.1.148	1.8742
2-C-methyl-D-erythritol 2,4-cyclodiphosphate synthase (MCS)	4.6.1.12	0.8282
4-hydroxy-3-methylbut-2-enyl diphosphate synthase (HDS)	1.17.7.1	5.4502
4-hydroxy-3-methylbut-2-enyl diphosphate reductase (HDR/IDS)	1.17.1.2	5.9418
acetyl-CoA acetyltransferase (AACT)	2.3.1.9	1.5835
hydroxymethylglutaryl-CoA synthase (HMGS)	2.3.3.10	3.0714
3-hydroxy-3-methylglutaryl-CoA reductase (HMGR)	1.1.1.34	20.4453
mevalonate kinase (MK)	2.7.1.36	2.2961
phosphomevalonate kinase (PMK)	2.7.4.2	1.6881
diphosphomevalonate decarboxylase (MVD)	4.1.1.33	2.3948
isopentenyl-diphosphate delta-isomerase (IPI)	5.3.3.2	1.9775
geranylgeranyl pyrophosphate synthetase (GGPS)	2.5.1.29	3.1042
squalene synthetase (SQS)	2.5.1.21	2.0913
cycloartenol synthase (CAS)	5.4.99.8	1.5239
squalene monooxygenase (SE)	1.14.13.132	1.5357
vascular plant one-zinc-finger transcription factor (VOZ)		1.3396

*VOZ*s are transcription factors that are highly conserved in land plant evolution [18, 19]. It has been shown to bind to the *cis*-element GCGTNx7ACGC, which belongs to the NAC subgroup VIII-2 [20]. *VOZ*s have been observed to be both positive and negative transcription factors of biotic and abiotic stress-response pathways, respectively, in *A. thaliana*. Although the *voz1voz2-2* mutant did not notably change in endogenous ABA content, the *voz1voz2* double mutant was inable to combat pathogens (*Pseudomonas syringae and Colletotrichum higginsianum*) due to low gene expression from defense-response genes [18]. This indicates that *VOZ*s are positive regulators in the SA and MJ signaling pathways in land plants. Therefore, we speculated that *VOZ* plays a postive role in the cucurbitacin pathway. A transcript from our gene set homologous to *VOZ* in *A. thaliana* and *Zea mays* was able to be identified with identities of 71.04% and 69.40% respectively.

We identified *HMGR* as containing the *VOZ cis*-element in its promoter region. In can be seen from Table 4 that *VOZ* expression was slightly up-regulated and *HMGR* was significantly up-regulated after MJ treatment. This provides evidence that *VOZ* transcription factors are a positive regulator that play a role, directly or indirectly, in the cucurbitacin pathway and biotic stress-response related genes.

The putative pathway also includes CYP450s and SAM-Mtases (a subset of the differentially expressed genes from Additional file 2: Table S1). CYP450s are one of the largest gene families in plants and catalyzes most oxidation steps in secondary metabolism such as in the biosynthesis of defense compounds, pigment, and antioxidants [21, 22]. Putatively, CYP450s may catalyze the conversion of cucurbitadienol. SAM-Mtases may also act on cucurbitadienol by catalyzing methylation, as it is known that many compounds with anti-microorganism functions have cucurbitadienol backbones activated by methylation [23]. We annotated 161 cytochrome P450s and 66 S-adenosyl-L-methionine-dependent methyltransferases (SAM-Mtases) in the *A. agallocha* genome, of which, 66 CYP450s and 27 SAM-Mtases showed significant up-regulation. These genes can be considered candidate genes that are possibly involved in the cucurbitacin pathway. As well, we identified a small number of SAM-Mtases that contained the *VOZ cis*-element, though their expression was not observed to be significantly up-regulated.

**Figure 5:**
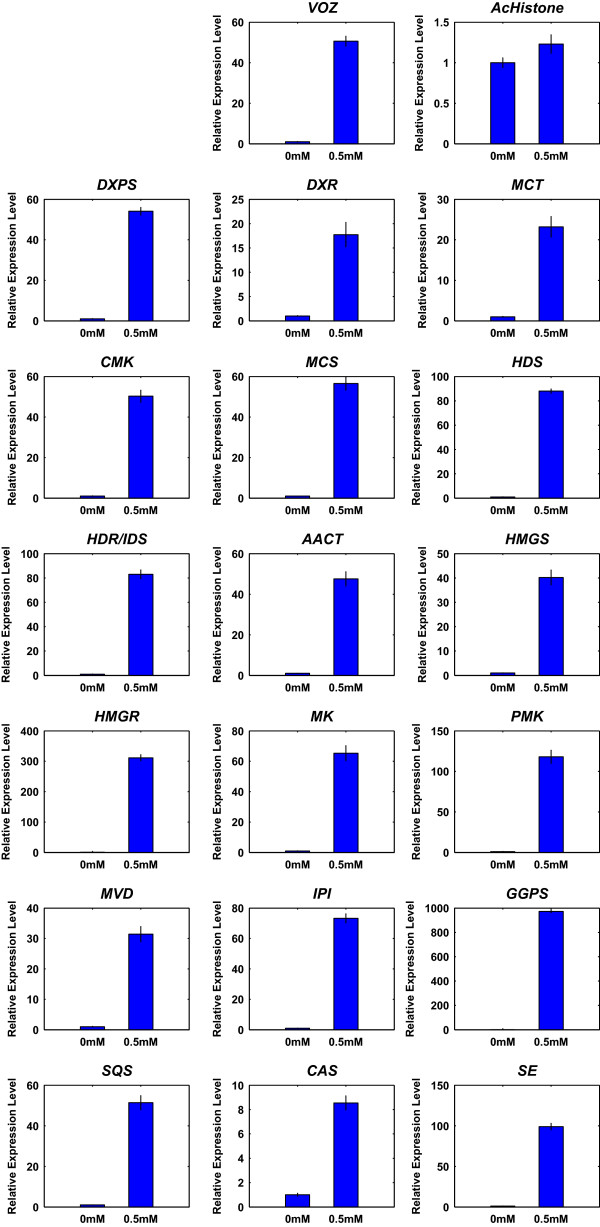
**qRT-PCR validation.** The qRT-PCR analysis results showing relative change in expression for each gene involved in the cucurbitacin pathway, as well as the *VOZ* transcription factor and the internal control *AcHistone*, after treatment with MJ.

The transcripts related to the cucurbitacin pathway and the *VOZ* transcription factor were validated using qRT-PCR (Figure 5). Our results are consistent with the expectation of cucurbitacin pathway genes being up-regulated in response to biotic stress. There is some disparity between qRT-PCR and RNA-seq values. However, this is likely due to differences in platform. The qRT-PCR primer sequences were designed for the 3′ ends of transcripts, which is highly stable, as compared to quantifying expression using the whole transcript with RNA-seq, which is typically not uniformly sequenced.

## Conclusion

Cucurbitacin, a compound of interest with medicinal value which exhibits anti-microorganism and anti-tumour properties was identified and observed to be abundant in *in vitro A. agallocha* agarwood. To better understand the genes and pathways related to cucurbitacin, a draft genome was assembled, from which, many genes and protein functions were able to be annotated and analyzed. The expression change for cucurbitacins was shown to be consistent with known responses of *A. agallocha* to biotic stress. As well, the DNA and RNA data was utilized to infer a putative pathway for cucurbitacin E and I in *A. agallocha* and a set of homologous genes in *A. thaliana* related to cucurbitacin bio-synthesis was presented. The *VOZ* transcription factor, a positive regulator of biotic stress-response pathways was identified as well as a putative regulation target among the genes related to the cucurbitacin pathway, *HMGR*, in which the *VOZ cis*-element was present in its promoter. The draft genome from this study can provide a resource for the investigation of secondary metabolite pathways not only in Aquilaria trees, but also in other non-model medicinal plants. The confirmation that cucurbitacins can be obtained from *in vitro* materials with a relatively high yield has positive implications with regards to plant factories to save on costs and time, as well as limiting the effects of research on this endangered species in the wild.

## Methods

### Plant materials for DNA and RNA extraction

All *in vitro* plant materials were grown under long-day conditions (16 h of light, 8 h of darkness) at 25°C. A plant regeneration system from shoot tips into *in vitro* plants was created using a tissue culture process similar to the processes described by He et al. [14]. Bio-assays were performed on the callus, *in vitro* shoot (2 months), and *in vitro* plant (4 months). DNA was extracted from 1 g of *in vitro* materials using the Plant Genomic DNA MiniKit (Maestrogen) following the manufacturer’s instructions. The DNA samples were sent to BGI for sequencing using HiSeq 2000. MJ treatment was performed according to the process described by Kumeta and Ito [4] where MJ was dissolved in DMSO to a concentration of 300 mM and then added to the culture at final concentrations of 0 mM, 0.1 mM, 0.2 mM, 0.5 mM, and 0.7 mM. RNA was extracted from 1 g of *in vitro* 0 mM and 0.5 mM MJ treated samples using RNeasy Plant MiniKit following the protocol prescribed by the manufacturer.

### LC-ESI-MS

*In vitro* materials were ground with liquid nitrogen and mixed with 1 mL of methanol. Supernatant was collected by centrifugation (12000 rpm, 1 min). The LC-ESI-MS system consisted of an ultra-performance liquid chromatography system (Ultimate 3000 RSLC, Dionex) and an electrospray ionization source of quadrupole time-of-flight mass spectrometer (maXis HUR-QToF system, Bruker Daltonics). The autosampler was set at 4°C. Separation was performed with reversed-phase liquid chromatography on a BEH C8 column (2.1 × 100 mm, Walters). The elution started from 99% mobile phase A (0.1% formic acid in ultrapure water) and 1% mobile phase B (0.1% formic acid in ACN), held at 1% B for 1.5 min, raised to 60% B in 6 min, further raised to 90% in 0.5 min, and then lowered to 1% B in 0.5 min. The column was equilibrated by pumping 1% B for 4 min. The flow rate was set to 0.4 mL/min with an injection volume of 5 μL. LC-ESI-MS chromatogram were acquired under the following conditions: capillary voltage of 4500 V in positive ion mode, dry temperature of 190°C, dry gas flow maintained at 8 L/min, nebulizer gas at 1.4 bar, and acquisition range of m/z 100–1000.

### Genome assembly

Five DNA libraries were constructed for a total of 144.3 Gb, which represents approximately 196X sequencing coverage (see Table 1) consisting of one paired-end library and four mate-pair libraries with various fragment lengths. First, the DNA paired-end read library was filtered according to base-call quality (25 of the first 35 bases from the 5′ end must be better than a quality score of 30 for read retention), removing sequence reads which contained ambiguous base-calls, and low complexity (sequences where 85% or more of the bases consist of the same nucleotide). The *A. agallocha* genome was assembled using String Graph Assembler (SGA) [24]. First, SGA was used to assemble the paired-end reads (kmer length of 41 for error correction, 65 and 79 for the minimum overlap and overlap lengths respectively) into contigs. Then, SSPACE [25] was used to construct scaffolds from the four mate-pair libraries (trimmed to 35 bp remaining at the 5′ end), which were utilized sequentially, from smallest to largest fragment size. Following scaffolding, gap closing was performed using GapFiller [26]. Sequences under 800 bp in length were excluded from the final assembly and subsequent analysis.

### Gene annotation

Repeat masking was performed on the assembled genome using RepeatMasker [27] and RepeatModeler [28] along with the TIGR plant repeat database [12] and Repbase (2012/04/18). RNA-seq reads were aligned to the repeat masked genome and potential transcripts were assembled using TopHat followed by Cufflinks [29]. The RNA-seq alignment results and assembled transcript sequences were used to generate extrinsic data for the gene prediction tool Augustus [30], which was used to predict gene models and transcripts from the draft genome assembly. The quality of the gene prediction was checked by performing both reference based transcript assembly with the draft genome using TopHat and Cufflinks as well as a *de novo* assembly using Velvet [31] followed by Oases [32]. For the reference based assembly, RNA-seq reads were aligned to the draft genome using TopHat, after which Cufflinks was used to assemble transcripts. The Cufflinks assembled transcripts were checked against the predicted transcripts from Augustus using blastn. The Velvet assembled transcripts were aligned to the draft genome using TopHat and blat. The regions in the draft genome which were able to be mapped by Velvet assembled transcripts were overlapped with Augustus predicted exon regions in order to determine how many exon locations were successfully predicted by Augustus. Transcripts from predicted gene-models were aligned against the NCBI non-redundant set of proteins using blastp (E-value 1E^-5^) to find homologues. The best alignment for each transcript was retained as annotation. Functional classification for the set of annotated transcripts was performed using the webserver Panther and its GO gene analysis tool [33].

### Differential expression

RNA-seq reads for the 0 mM and 0.5 mM MJ treatment conditions were individually aligned to the set of annotated transcripts using BWA [34]. For each condition, quantification of transcript expression was performed by using eXpress [35] to calculate the fragments per kilobase per million (FPKM) for each transcript. The fold change (0.5 mM/0 mM) was calculated for each transcript from the FPKM values. A transcript was denoted up-regulated or down-regulated if the log2 fold change was greater than 1 or less than −1, respectively, otherwise, a transcript was denoted non-differentially expressed. The fold change at the gene level was obtained by averaging the fold change ratios of all transcripts belonging to the same gene, as determined by annotation using blastp [36]. In other words, the fold change for transcripts assigned to the same *A. thaliana* gene ID was averaged to obtained a gene level fold change. Functional classification for the set of differentially expressed transcripts was performed using the webserver Panther and its GO gene analysis tool [33].

### Putative cucurbitacin E and I pathway

From the differential expression analysis, transcripts which exhibited expression (FPKM > 0) in both 0 mM and 0.5 mM MJ treatment conditions were annotated with *A. thaliana* proteins from TAIR as well as UniProt enzymes for EC classifications using blastp (E-value 1E^−5^). The resulting set of annotated transcripts was used to infer a putative cucurbitacin E and I pathway by referring to the mevalonate pathway of *A. thaliana* from KEGG [15].

The promoter regions of genes related to the cucurbitacin pathway was assayed for the *VOZ cis*-element, GCGTNx7ACGC. A promoter region was denoted as 5,000 bp upstream from the gene TSS. A sequence motif search for the *VOZ cis*-element was performed in the promoter regions using Perl.

### qRT-PCR validation

Validation of the curcubitacin I pathway transcripts as well as the transcription factor *VOZ* found in *A. agallocha* was performed using qRT–PCR analysis. The 0 mM and 0.5 mM MJ treated RNA samples were extracted from 1 g of four month old *in vitro A. agallocha* shoots using RNeasy Plant MiniKit following the protocol prescribed by the manufacturer. Primers pairs were designed for each transcript (Additional file 3: Table S2) with the ABI Prism 7500 sequence detection system (Applied Biosystems). Each primer pair was used to amplify the respective cDNA fragments using a cycling profile consisting of 58°C for 2 min, 95°C for 10 min, and 40 cycles of 95°C for 15 s and 60°C for 1 min. The relative gene expression was determined by the comparative CT method, 2^−ΔCT^ (ΔC_T_ = C_T_, gene of interest – C_T_, control gene), using *AcHistone* as the internal control [5]. Four independent biological repeats were performed for each assay where the final expression value is the mean expression of the repeats.

### Availability of supporting data

All supporting data used in this study is publicly available at NCBI under BioProject: PRJNA240626 (http://www.ncbi.nlm.nih.gov/bioproject/PRJNA240626). Specifically, the genome assembly can be obtained under GenBank Assembly ID: GCA_000696445.1 (http://www.ncbi.nlm.nih.gov/assembly/GCA_000696445.1). The transcriptome sequences are under SRA accessions: SRX550129 (http://www.ncbi.nlm.nih.gov/sra/SRX550129), SRX540116 (http://www.ncbi.nlm.nih.gov/sra/SRX540116). As well, our *A. agallocha* specimen has been submitted to a herbarium at Herbarium, Research Center for Biodiversity, Academia Sinica, Taipei (HAST) under the accession number 137059 (http://www.hast.biodiv.tw/specimens/SpecimenDetailE.aspx?specimenOrderNum=137059).

## Electronic supplementary material

Additional file 1: Figure S1: Identification of Cucurbitacin I (formula weight: 514.65 g) with LC-ESI-MS. Red represents the shoot tip sample mixed with cucurbitacin I standard. Green represents the shoot tip sample. **Figure S2.** Identification of Cucurbitacin E (formula weight: 556.69 g) with LC-ESI-MS. Red represents the shoot tip sample mixed with cucurbitacin E standard. Green represents the shoot tip sample. **Figure S3.** Genome size of *A. agallocha* by flow cytometry. The haploid genome size of *A. agallocha* was approximately 0.604-fold of that of the reference standard (CEN Singlet; 2C = 2.5 pg DNA, 1 pg = 978 Mb). (DOC 5 MB)

Additional file 2: Table S1: The set of 4,827 differentially expressed genes. (CSV 218 KB)

Additional file 3: Table S2: Gene specific primers for real-time PCR analysis of gene expression. (DOC 34 KB)
