# Supplementary material for: Identification of cucurbitacins and assembly of a draft genome for Aquilaria agallocha
Source: BMC Genomics. 2014 Jul 9;15(1):578. doi: 10.1186/1471-2164-15-578 (PMC4108785; doi:10.1186/1471-2164-15-578)
Supplement: Supplementary file 1 — Additional file 1: Figure S1: Identification of Cucurbitacin I (formula weight: 514.65 g) with LC-ESI-MS. Red represents the shoot tip sample mixed with cucurbitacin I standard. Green represents the shoot tip sample. Figure S2. Identification of Cucurbitacin E (formula weight: 556.69 g) with LC-ESI-MS. Red represents the shoot tip sample mixed with cucurbitacin E standard. Green represents the shoot tip sample. Figure S3. Genome size of A. agallocha by flow cytometry. The haploid genome size of A. agallocha was approximately 0.604-fold of that of the reference standard (CEN Singlet; 2C = 2.5 pg DNA, 1 pg = 978 Mb). (DOC 5 MB) [file 12864_2013_6271_MOESM1_ESM.doc]

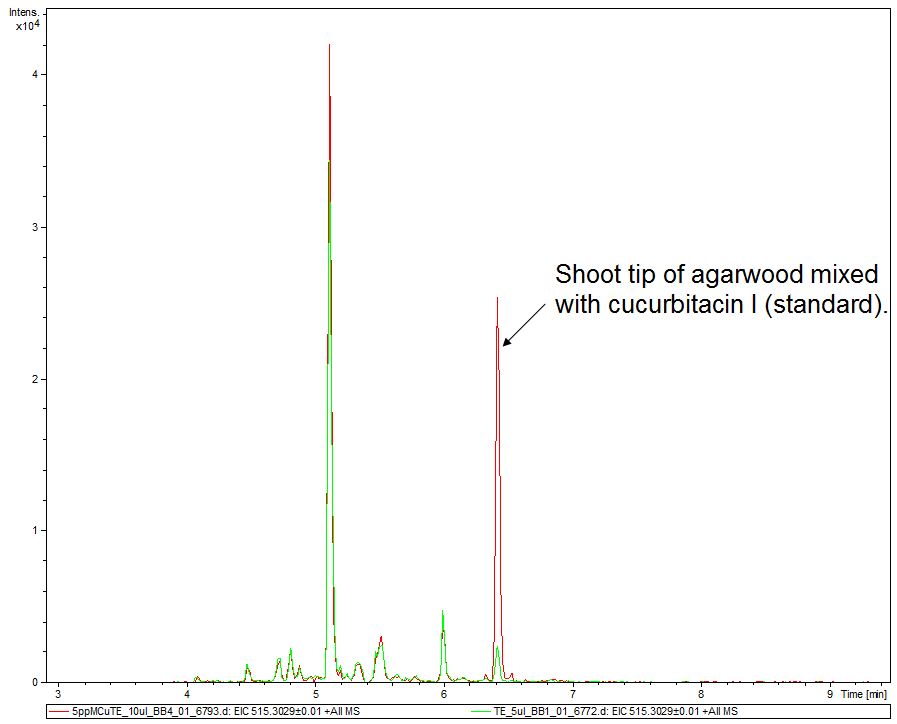


**Supplementary Figure S1.** Identification of Cucurbitacin I (formula weight: 514.65 g) with LC-ESI-MS. Red represents the shoot tip sample mixed with cucurbitacin I standard. Green represents the shoot tip sample.


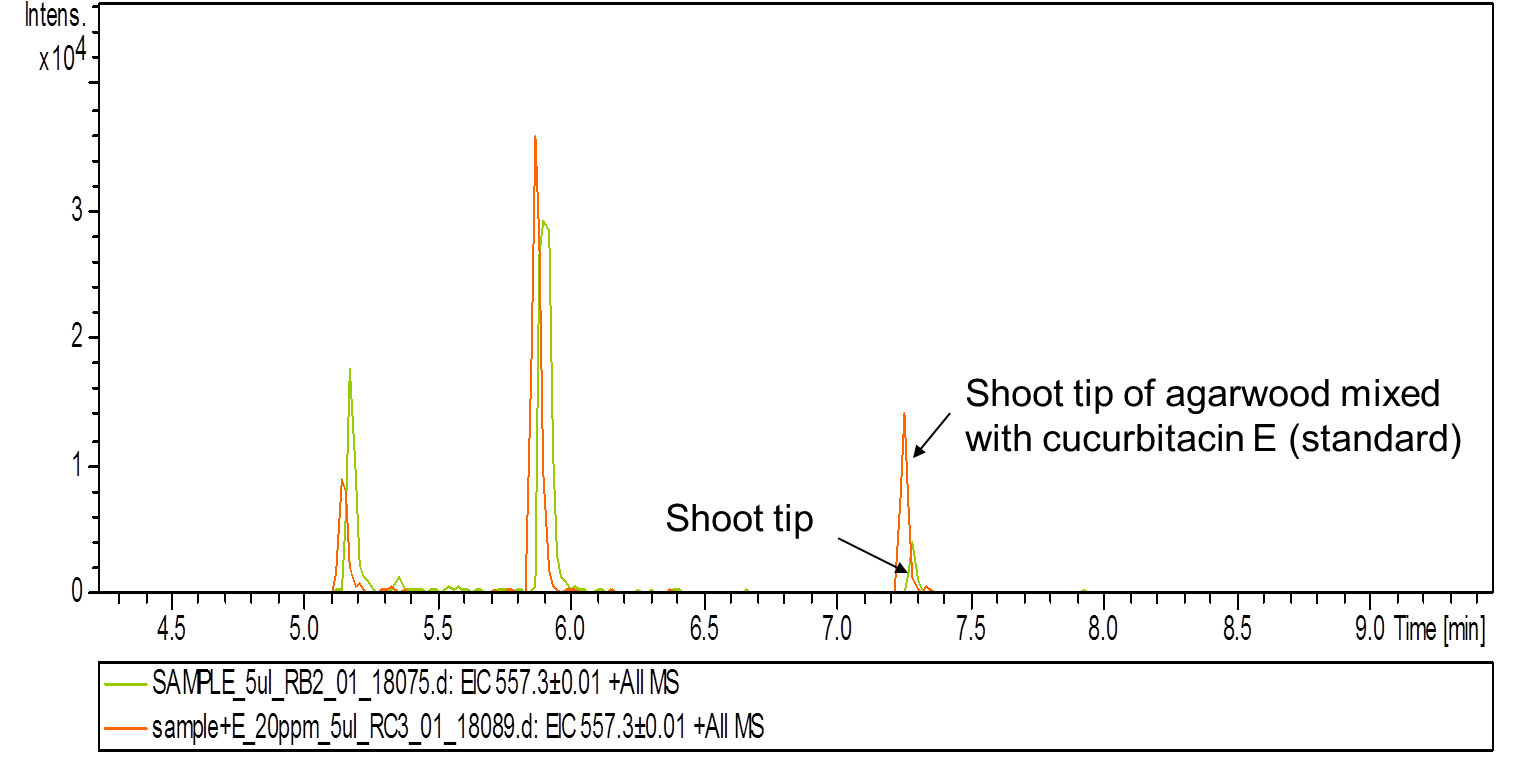


**Supplementary Figure S2.** Identification of Cucurbitacin E (formula weight: 556.69 g) with LC-ESI-MS. Red represents the shoot tip sample mixed with cucurbitacin E standard. Green represents the shoot tip sample.


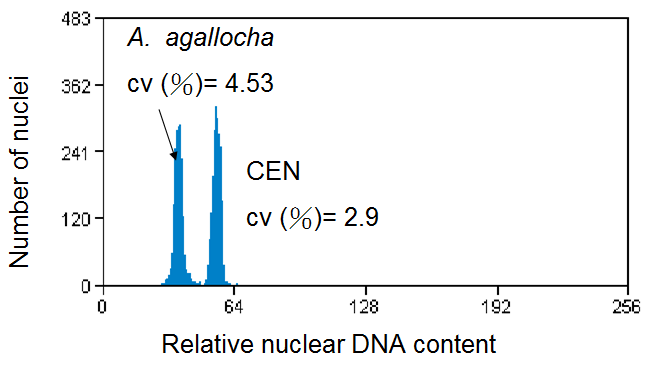


**Supplementary Figure S3.** Genome size of *A. agallocha* by flow cytometry. The haploid genome size of *A. agallocha* was approximately 0.604-fold of that of the reference standard (CEN Singlet; 2C = 2.5 pg DNA, 1 pg = 978 Mb).
