# Supplementary material for: Identification of cucurbitacins and assembly of a draft genome for Aquilaria agallocha
Source: BMC Genomics. 2014 Jul 9;15(1):578. doi: 10.1186/1471-2164-15-578 (PMC4108785; doi:10.1186/1471-2164-15-578)
Supplement: Supplementary file 3 — Additional file 3: Table S2: Gene specific primers for real-time PCR analysis of gene expression. (DOC 34 KB) [file 12864_2013_6271_MOESM3_ESM.doc]

**Supplementary Table S2.** Gene-specific primers for real-time PCR analysis of gene expression

*Internal Control: AcHistone*

forward primer: 5'-GTACCGCTACCGGAGGGAAGTTGAAGA-3'

reverse primer: 5'-CTTCTTGGGCGACTTGGTAGCCTTGGT-3'

*1.1.1.34* , AT1G76490.1*:*

forward primer: 5'- TGCCGATTTGAAGCAGTACTTG -3'

reverse primer: 5'- GCCACGGTCTCAAAATTCATG -3'

1.1.1.267, AT5G62790.1*:*

forward primer: 5'- ATGTCATGGCCAGAGAGGATCT -3'

reverse primer: 5'- CAAGACGAGGCCAGGTTATTTC -3'

1.14.13.1, AT1G58440.1*:*

forward primer: 5'- AAGTTCGCTGCTTGGTCGATAT -3'

reverse primer: 5'- TGGCAACAGGAGGTAGTTTCTG-3'

1.17.1.2, AT4G34350.1*:*

forward primer: 5'- TTGTTGTTGGTGGCTGGAAC -3'

reverse primer: 5'- CCCATGATCCTCAGCAATCTC -3'

1.17.7.1, AT5G60600.1*:*

forward primer: 5'- ATGGAGTATGCGACTGACGCTT -3'

reverse primer: 5'- CCAGCGGCCATGATCTTTTATA -3'

2.2.1.7, AT4G15560.1*:*

forward primer: 5'- TGCAATGGATCGAGCTGGTT -3'

reverse primer: 5'- CCACAGTGTGTTGGTCCATCAG -3'

2.3.1.9, AT5G48230.1*:*

forward primer: 5'- AATGTGAATGGCGGAGCTGTA -3'

reverse primer: 5'- TCCACTGCAACCCAAAGGAT-3'

2.3.3.10, AT4G11820.2*:*

forward primer: 5'- ACGGAGGTGGACTCCAAGTACA -3'

reverse primer: 5'- CAGCGTTCTCCTCACCTGTCTT -3'

2.5.1.21, AT4G34640.1*:*

forward primer: 5'- ACGGACAGAGCCAAACTACACA -3'

reverse primer: 5'- GCCAACAGAATGAAGACCACAA -3'

2.5.1.29, AT4G36810.1*:*

forward primer: 5'- GTATTCGGTGAGGATATCGCCG -3'

reverse primer: 5'- GTGCTCGAAGGCAAAAGAGAGG -3'

2.7.1.36, AT5G27450.1*:*

forward primer: 5'- TCAAAATGCTCATCACCGACAC -3'

reverse primer: 5'- CCAGCGACTAATGCTTTCGTG -3'

2.7.1.148, AT2G26930.1*:*

forward primer: 5'- CCCCAGATCCTCCACAATTTGT -3'

reverse primer: 5'- AAAACACCTCGCGGTAATCCTC -3'

2.7.4.2, AT1G31910.1*:*

forward primer: 5'- AAGGCAGTGATCCACGAAACC -3'

reverse primer: 5'- CGGACCGAAGAGATAGCAGTTG -3'

2.7.7.60, AT2G02500.1*:*

forward primer: 5'- GAAATGCAAACACCTCAGGTGA -3'

reverse primer: 5'- CAAAGCCTTTCTTGAGCAGCTC -3'

4.1.1.33, AT3G54250.1*:*

forward primer: 5'- TGGAGGAAGCCATAAAAGATCG -3'

reverse primer: 5'- CACGTCAATTGTGCAAAGGAAG -3'

4.6.1.12, AT1G63970.1*:*

forward primer: 5'- CGTAAACCTCAAGGCAAAAACG -3'

reverse primer: 5'- CGCTGCAATACTTCGATTCTCC-3'

5.3.3.2, AT3G02780.1*:*

forward primer: 5'- ATTAAGCTGTCGCCCTGGTTC -3'

reverse primer: 5'- CAGATGGTCCCACCACTTGAA -3'

5.4.99.8, AT2G07050.1*:*

forward primer: 5'- CCACTGCATCATGCAGCAA -3'

reverse primer: 5'- CTGAGGAAAATCTCCCGTCTCC-3'

VOZ, AT1G28520.1*:*

forward primer: 5'- GGCATGAATCAAGGAAGCAAGT -3'

reverse primer: 5'- CCAGTCTTTTCTGCCCTCCATA -3'
